# Determination of Very Low Level of Free Formaldehyde in Liquid Detergents and Cosmetic Products Using Photoluminescence Method

**DOI:** 10.1155/2016/1720530

**Published:** 2016-08-21

**Authors:** Ali Gholami, Atefeh Mohsenikia, Saeed Masoum

**Affiliations:** Department of Analytical Chemistry, Faculty of Chemistry, University of Kashan, Kashan 87317-53153, Iran

## Abstract

Formaldehyde is commonly used in detergents and cosmetic products as antibacterial agent and preservative. This substance is unfavorable for human health because it is known to be toxic for humans and causes irritation of eyes and skins. The toxicology studies of this compound indicate risk of detergents and cosmetic formulations with a minimum content of 0.05% free formaldehyde. Therefore, determination of formaldehyde as quality control parameter is very important. In this study, a photoluminescence method was achieved by using 2-methyl acetoacetanilide. Also, the Box-Behnken design was applied for optimization of Hantzsch reaction for formaldehyde derivatization. The investigated factors (variables) were temperature, % v/v ethanol, reaction time, ammonium acetate, and 2-methyl acetoacetanilide concentration. The linear range was obtained from 0.33–20 × 10^−7^ M (1–60 *μ*g·kg^−1^) and the limit of detection (LOD) was 0.12 *μ*g·kg^−1^. The proposed method was applied for the analysis of Iranian brands of liquid detergents and cosmetic products. The formaldehyde content of these products was found to be in the range of 0.03–3.88%. Some brands of these products had higher concentration than the maximum allowed concentration of 0.2%. High recoveries (96.15%–104.82%) for the spiked dishwashing liquid and hair shampoo indicate the proposed method is proper for the assessment of formaldehyde in detergents and cosmetic products. The proposed methodology has some advantages compared with the previous methods such as being rapid, without the necessity of applying separation, low cost, and the fact that the derivatization reaction is carried out at room temperature without any heating system.

## 1. Introduction

Formaldehyde is a strong antibacterial and preservative that is utilized for controlling microbial growths in water containing solutions and organic materials. Formaldehyde is a gaseous and colorless molecule at room temperature and it is readily soluble in water. This compound is a strong antibacterial and very low cost preservative so producers use it in liquid detergents and cosmetic products instead of other preservatives. The hazard of formaldehyde has been reported which found enough evidence for the mutagenicity and carcinogenicity of this compound [1, 2]. Formaldehyde is recognized to be toxic for human beings. It is an irritating agent to the respiratory zone and eye which can cause allergic contact dermatitis or eczema (drying and reddening of the skin) at high concentration [3]. The formaldehyde and formaldehyde releasing preservative is widely used in surfactants, dishwashing liquids, cosmetic products particularly hair shampoos, and care products such as hair straightening products.

Toxicology of formaldehyde has been widely discussed and studies have indicated the highest risk for individuals with a daily contact with this substance [4, 5]. The use of formaldehyde as a preservative is allowed to a maximum concentration of 0.2% in detergent and cosmetic products (with the exception of nail polish for which a concentration up to 5% is allowed). Due to allergic potential of formaldehyde, liquid detergents and cosmetic products must be labeled with “contain formaldehyde” when there is a minimum content of 0.05% free of this substance [6, 7]. Because of the above reasons and the influence of formaldehyde in human bodies, various methods have been developed for determination of formaldehyde in some detergents and cosmetic products including high performance liquid chromatography (HPLC) [8–11], headspace solid phase microextraction by gas chromatography (GC), and isotope dilution mass spectrometry [12, 13], fluorimetry [14], and spectrophotometry [15, 16].

Various reagents have been proposed for derivatization of formaldehyde by spectrophotometry [17–21] and also fluorimetry [22–26]. However, spectrophotometric method is not sensitive enough for the analysis of real samples and sometimes subject to numerous interferences which are serious problems.

Li et al. examined several novel reagents for detection of formaldehyde based on the Hantzsch reaction which were benzoylacetone, N-methyl acetoacetamide, n-acetoacetyl-o-toluidine, and acetoacetanilide (AAA) used in fluorimetry [27].

In this study, 2-methyl acetoacetanilide was proposed for determination of the Hantzsch reaction by photoluminescence method. The proposed method is based on the Hantzsch reaction, which involves the cyclization between 2-methyl acetoacetanilide and formaldehyde in the presence of ammonium acetate. The derivatization reaction of formaldehyde with 2-methyl acetoacetanilide is shown in Scheme S1 (see Supplementary Material available online at http://dx.doi.org/10.1155/2016/1720530).

The aim of this study is applying fluorescence spectroscopy to routine determination of formaldehyde in detergents and cosmetic products. Fluorescence spectroscopy as an analytical technique has the advantage of selectivity and sensitivity in comparison with other spectroscopic methods.

The derivatization reaction of formaldehyde by the proposed reagent is depending on several factors. Optimization of effective factors on experiment usually was done by classical method. The classical method is based on one factor at a time. Although this method may be efficient in some experiments, it is not effective while there are several independent variables and interaction influences affecting the responding factors. Design of experiments (DOE) is required in this situation. Design of experiments (DOE) is a powerful technique used for discovering a set of variables (or factors) which are most important to the process or experiment and then determining what levels of these factors must be kept to optimize the process performance [28].

Optimization strategy sets several experiments that can determine all factors and probable interactions between these independent variables. When the responses are influenced by several factors, response surface methodology (RSM) is a useful tool of modeling with a collection of statistical and mathematical techniques. The Box-Behnken design is a second-order design under RSM that requires 3 levels of each factor; it is reliable, less laborious, efficient, cost-effective, providing sufficient data on the influence variables and decreasing the number of experimental trials and overall experiment error with a minimum number of experiments [29].

## 2. Experimental Section

### 2.1. Chemicals and Reagents

All chemicals used in this research were ultra pure. Distillation water was used for the preparation of all solutions. Stock solution of 2-methyl acetoacetanilide (0.3 M) was prepared by dissolving 5.73 g of 2-methyl acetoacetanilide (Aldrich Pure Chemicals, Germany) in 50 mL of ethanol and diluting it to 100 mL with distillation water. An ammonium acetate stock solution was prepared by dissolving 54.00 g of ammonium acetate (Merck Pure Chemicals, Germany) in the water and diluting it to 100 mL with the purified water. The pH of ammonium acetate was adjusted as 7.5 by addition of 0.1 M NaOH. A 0.10 M standard solution of formaldehyde was prepared by diluting 0.9 mL of 30.0% formaldehyde solution to 100 mL with distillation water, followed by an accurate concentration determination using the iodometric method before analysis since formaldehyde is volatile. Formaldehyde reacts with potassium iodide solution and iodine is back-titrated with standard solution of sodium thiosulphate in the presence of starch indicator. The working standard solutions were daily prepared by accurate dilution of the standard stock solution. For interference testing, the following compounds were used: sodium chloride, iron (III) nitrate, nickel, lead and cadmium (II) nitrate, calcium and magnesium (II) chloride, benzaldehyde, propionaldehyde, and acetaldehyde.

### 2.2. Instrumentation

A photoluminescence spectrometer LS55 (PerkinElmer, UK) was employed for spectral measurements. A pH meter model 691 (Metrohm, Swiss), heater stirrer (Gerhardt, Germany), and analytical balance model R160P (Sartorius, Germany) were applied. ^1^HNMR spectra were recorded on Bruker avance-400 MHZ spectrometers in the presence of tetra methyl silane as internal standard.

### 2.3. Experimental Design and Method of Analysis

Derivatization reaction of formaldehyde is depending on several factors. Box-Behnken design contains numbers of design points and a repeated center point for calculating of error. Box-Behnken design does not contain an embedded factorial design like the central composite design and instead of design points there are the midpoints and it requires 3 levels (−1, 0, and +1) for each factor. The Box-Behnken design can be considered a highly fractionalized three-level factorial design where the treatment combinations are the midpoints of edges of factor levels and the center point. These designs are rotatable (or nearly rotatable) and require three levels each under study factors. Box-Behnken design can fit full quadratic response surface models that it does not contain an embedded factorial or fractional factorial design and does not contain axial points so all design points are sure to be within safe operating limits. In this design, the treatment combinations are at the midpoints of edges of the process space and at the center. The designs have limited capability for orthogonal blocking compared to the central composite designs.

The advantages of the Box-Behnken design over other response surface designs are as follows: (a) it needs fewer experiments than central composite design (less points); (b) in contrast to central composite designs, it has only three levels; (c) it is easier to arrange and interpret than other designs; (d) it avoids combined factor extremes since midpoints of edges of factors are always used [30]. In this work, because of derivatization reaction of formaldehyde depending on five factors in three levels, we employed the response surface method and Box-Behnken design to optimize the independent factors that are effective on the response.

In order to reduce the effects of unexplained variability in the real responses due to irrelevant parameters, the experiments were done in randomized order. The independent factors and their ranges were selected based on preliminary experiment results. Then, a second-order quadratic equation was fitted to the data by multiple regression procedure. The generalized response surface model for a five-factor system is shown by

(1) Yi=α0+α1x1+α2x2+α3x3+α4x4+α5x5+α11x12+α22x22+α33x32+α44x42+α55x52+α12x1x2+α13x1x3+α14x1x4+α15x1x5+α23x2x3+α24x2x4+α25x2x5+α34x3x4+α35x3x5+α45x4x5,

where *Y*_*i*_ is the predicted response, *x*_*i*_ values (*i* = 1, 2, 3, 4, and 5) are the independent variables, *α*_0_ is the intercept (constant), *α*_1_, *α*_2_, *α*_3_, *α*_4_, and *α*_5_ are linear coefficients, *α*_11_, *α*_22_, *α*_33_, *α*_44_, and *α*_55_ are squared coefficients, *α*_12_, *α*_13_, *α*_14_, *α*_15_, *α*_23_, *α*_24_, *α*_25_, *α*_34_, *α*_35_, and *α*_45_ are interactions coefficients.

In this work, five studied factors consist of reaction time (*x*_1_), % v/v ethanol (*x*_2_), ammonium acetate concentration (*x*_3_), 2-methyl acetoacetanilde concentration (*x*_4_), and temperature (*x*_5_) on the analytical signal. Minitab14 as a statistical software package was used for data processing, studying linear terms, squared terms, and interaction between the variables.

## 3. Results

### 3.1. Optimization by Response Surface Method

A Box-Behnken design was applied to estimate the correlation of the five independent factors, with three levels for each factor. In this study, 46 experiments were employed to fit a second-order polynomial model. The variables *x*_1_–*x*_5_ were studied to optimize the fluorescence intensity. The name of independent effective factors and their coded levels scheme of Box-Behnken design are shown in Tables 1 and S1, respectively. The optimum values were calculated for effective parameters on derivatization reaction for 50 *μ*g·kg^−1^ of formaldehyde. The optimum value of each variable was calculated through codes and their corresponding values to be encoded (actual) with Minitab14 software (Table 2). Additionally, the optimum response values of each factor can be obtained by the graphical analysis of the surface related to each equation obtained by Minitab software (Figures S1 and S2). The effects of efficient parameters, regression coefficients, and the associated standard errors for derivatization reaction of formaldehyde are shown in Table S2.

### 3.2. Calibration Graph and Analytical Parameters

The excitation and emission spectra were obtained with 2-methyl acetoacetanilide in the presence and in the absence of formaldehyde. The florescence intensity was enhanced greatly in the presence of formaldehyde. This result indicates that a new florescence compound was formed. The maximum wavelengths of excitation and emission were 360 and 460 nm, respectively.

**Figure 1:**
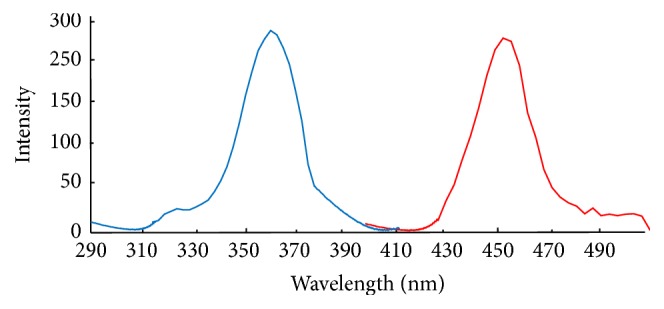
Maximum excitation and emission wavelength for product of formaldehyde derivatization with 2-methyl acetoacetanilide.

Figure 1 shows maximum excitation and emission wavelength for product of formaldehyde derivatization with 2-methyl acetoacetanilide. The calibration curve for determination of formaldehyde was constructed under the optimum condition summarized in Table 2.

**Figure 2:**
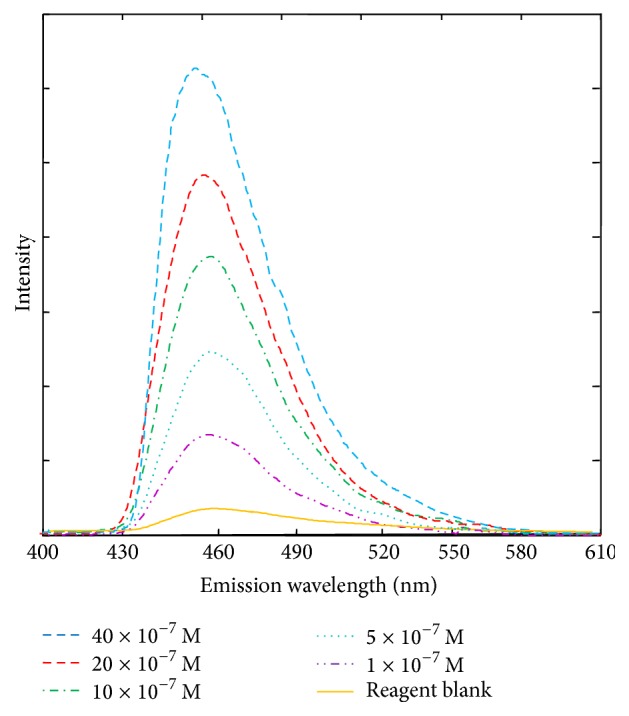
Emission spectra of 0–20 × 10^−7^ M of formaldehyde with 2-methyl acetoacetanilide.

For fluorometric measurements, emission spectra of 0–20 × 10^−7 ^M of formaldehyde were recorded (Figure 2).

The fluorescence response was linear in the formaldehyde concentration range of 0.33−20 × 10^−7 ^M (1–60 *μ*g·kg^−1^). The equation of the calibration graph for 0.33–20 × 10^−7 ^M was expressed as *Y* = 40.12*x* + 42.12, where *Y* is the fluorescence intensity and *x* is the formaldehyde concentration, with a correlation coefficient of 0.997.

As ^1^HNMR spectroscopy is a perfect method for characterization of chemicals, this method was used for characterization of the Hantzsch product. All protons of Hantzsch product were correctly determined in the ^1^HNMR:

 
*δ* = 2.4 ppm: 12H (4 CH_3_).
 
*δ* = 3.6 ppm: 2H (CH_2_).
 
*δ* = 5.2 ppm: 1H (NH amine).
 
*δ* = 7.0–7.5 ppm: 8H (aromatic rings).
 
*δ* = 7.9 ppm: 2H (NH amide).

According to the ^1^HNMR data (Figure S3), we conclude that the Hantzsch product was successfully synthesized and purified.

In the ^1^HNMR spectra, the signals of methyl groups joined to the double bond and benzene ring appear as signals at *δ* = 2.4 and 2.27 ppm, respectively. Singlet splitting related to methylene groups located at *δ* = 3.6 ppm. The NH protons related to amino group appears in *δ* = 5.16 ppm (Figure S4), while NH signal related to amino groups is shown in 7.86 and 7.87 ppm, respectively. The signals about *δ* = 7.0–7.5 ppm assigned by proton of CH-CH of aromatic rings in the product spectrum (Figure S5).

The limit of detection is calculated as the concentration corresponding to three times of the baseline noise (*S*/*N* = 3). The limit of detection was 4 × 10^−9 ^M (0.12 *μ*g·kg^−1^).

### 3.3. Application of the Proposed Method for Determination of Formaldehyde in Real Samples

The proposed methodology was applied for the analysis of Iranian brands of liquid detergents and cosmetic products. some of these products contained detectable amounts of formaldehyde that were higher than the maximum allowed concentration of 0.2%. The formaldehyde content of these products was found to be in the range 0.03–3.88%. The results were shown in Table 3.

For evaluation of the precision and accuracy of the proposed method, spiking experiments were performed. In Table 4, we spiked 50 mg·Kg^−1^ of formaldehyde to surfactant, hair shampoo, body shampoo, and hand cleaner which have no free formaldehyde. After spiking experiments, recovery factors were then calculated according to the following equation (the results are shown in Table 4):

(2) $$\begin{array}{c} \text{Recovery factor}=100\times \frac{\left(C_{Recovered}\right)}{C_{True}}, \end{array}$$

where *C*_Recovered_ is the measured concentration after adding known concentration of the analyte to the real sample and *C*_True_ is the expected concentration [31].

In Table 5, we spiked 3, 5,  and 8 *μ*g·Kg^−1^ of formaldehyde to dishwashing liquid 1 and hair shampoo 1 that contain formaldehyde. After spiking experiments, recovery factor and relative standard errors were calculated (Table 5).

### 3.4. Stability of Formaldehyde after a Few Months

The use of formaldehyde as a preservative in liquid detergents such as dishwashing liquids and cosmetic products such as hair and body shampoos and shower gel is allowed to a maximum concentration of 0.2%, that is, 600 mg·Kg^−1^. Considerable decreasing in the content of formaldehyde occurs after a few months in detergents and cosmetic products.

### 3.5. Interferences from Foreign Substances

#### 3.5.1. Spectral Interferences

The effect of various spectral interferences possibly present in the real detergent samples was investigated. Detergents and cosmetic products such as hair and body shampoos contain water (about 70%), surfactants as a cleaning and foaming agent (about 25%), and additives (about 5%) such as colors, fragrances, and other compounds. Three-dimensional florescence spectrum of a shampoo sample was recorded after derivatization of formaldehyde (Figure S6). To generate three-dimensional fluorescence, excitation wavelengths between 200 and 400 nm with 10 nm intervals and emission wavelengths between 250 and 500 nm with 0.5 nm intervals were used.

#### 3.5.2. Ion Interferences

The tolerable concentrations are defined as the concentration of foreign species causing less than 5% relative error. The influences of foreign species were examined by adding a certain amount of species in 1.0 × 10^−6 ^M HCHO solution. The results are shown in Table 7.

## 4. Discussion

Derivatization reaction of formaldehyde is depending on several factors. The Hantzsch reactions with some diketones analogues reagents are usually slow and therefore detection reactions must be carried out at higher temperature than room temperature. Experimental results demonstrated that the reaction could proceed better in some organic solvents. A comparison between ethanol, methanol, and acetone was examined. The results imply that higher efficiency could be obtained in ethanol solution medium. The effect of reaction temperature on signal intensity in reaction of 2-methyl acetoacetanilide with formaldehyde was examined by varying it from 20 to 60°C. The results obtained are shown in Figure S7 that, in the temperature over 30, the fluorescence intensity is decreased.

The experimental design results show that 27°C is the optimum temperature; thus, above 28°C, the intensity gradually decreased with increasing temperature. However, for convenient operation, 25°C (room temperature) was selected.

In the reaction of formaldehyde with the 2-methyl acetoacetanilide reagents, pH of the reagent solution is very important for the reaction efficiency. The preliminary examination showed that the fluorescence intensity of product of formaldehyde with 2-methyl acetoacetanilide in acetate buffer was much higher than that in phosphate buffer. Replacement of ammonia by ammonium acetate allowed the efficient synthesis of Hantzsch's compounds under mild conditions. Hence, in this work, ammonium acetate was chosen as a buffer. The effect of pH on the sensitivity was investigated in the range of pH 5.0–8.0 using ammonium acetate as buffers. The pH was adjusted with acetic acid and NaOH solution. The results obtained are shown in Figure S8, which indicates that, in the pH range over 6.5–7.5, the fluorescence intensity is increased, and above pH 7.5, the fluorescence intensity becomes decreased. From these results, the pH of 7.5 was chosen for further experiments. In all experiments, the pH of ammonium acetate was adjusted as 7.5.

Despite the official regulation, high contents of formaldehyde in detergent and cosmetic products have been reported in Iranian brand of these products but producers have labeled their products “formaldehyde free.” Our results confirmed that the risk of cosmetic and detergent formulations with formaldehyde above 0.2% is not negligible, as these products may facilitate considerable exposure of formaldehyde for consumers.

After spiking experiments, recovery factors were calculated. Comparison of the predicted concentrations and recoveries provided by the proposed method shows a good predictive ability towards the spiked liquid detergents and cosmetic products for determination of formaldehyde with 2-methyl acetoacetanilide by photoluminescence method.

The stability of formaldehyde has been determined in dishwashing liquid 1 and hair shampoo 1. The media of cosmetic and detergent products contain water and organic compounds such as surfactants and additive compounds such as colors and fragrances that bacteria could grow easily. It is evident from Table 6 which considerable decreasing occurs in free formaldehyde content after a few months of controlling microbial growths in water containing solutions and organic material.

The effect of various spectral interferences possibly present in the real detergent samples was investigated. Three-dimensional florescence spectrum of a shampoo sample was recorded after derivatization of formaldehyde (Figure S6). In Figure S6, there is no overlap spectrum between compound in shampoo and product of formaldehyde derivatization. The results are not acceptable if there is spectral overlap between product of formaldehyde derivatization and other compounds in real samples. In order to remove spectral overlap, second-order calibration such as three-way methods must be used. These algorithms can be applied with great success to identify and quantify overlapped fluorophores. Due to the unique solution of three-way methods (e.g., parallel factor analysis), they can resolve overlapping signals into pure spectra and relative concentration profile. The influences of foreign species were examined. Sodium and chloride did not affect up to 100000-fold excess over formaldehyde. Calcium and magnesium did not affect up to 1000-fold excess over formaldehyde. Benzaldehyde did not affect up to 1000-fold excess over formaldehyde. Acetaldehyde and propionaldehyde did not affect up to 500-fold excess over formaldehyde. Nickel, lead, and cadmium did not affect up to 100-fold excess over formaldehyde did not interfere. Iron (III) interfered with determination of formaldehyde at a concentration ratio more than 50. These results indicate that the proposed method has good selectivity for determination of formaldehyde (Table 7).

In this research, a rapid, simple, and selective method was developed for determination of formaldehyde in detergents and cosmetic products. By spiking known concentration of formaldehyde to the real samples, the accuracy of the proposed methods was validated and recoveries of the spiked value were calculated. The proposed method minimizes an additional extraction step and consumption of expensive hazardous and environmentally unfriendly organic solvents and allows saving time compared with liquid and gas chromatography methods. In addition, the expanses of GC/MS systems are high, operation system is not easy and unavailable in many laboratories, and a separation step was needed to remove the possible matrix effect of detergents and cosmetic products samples.

The reaction of formaldehyde with 2-methyl acetoacetanilide was done in room temperature in comparison with other regents such as acetylacetone or fluoral P that need high temperature and longer reaction time.

The proposed methodology was applied for the analysis of different Iranian commercial brands of liquid detergents and cosmetic products. Some brands of these products have measurable amounts of formaldehyde that were higher than the maximum allowed concentration of 0.2%. The obtained investigation suggests that consumer and user information are required to warn about the highest risks of using detergents and cosmetic products with high formaldehyde content because of allergenic potential and evidence for carcinogenicity of this substance. The results in Table 8 show comparison between the proposed method and other methods.

## Supplementary Material

Despite official regulations, the illegal use of formaldehyde in detergents and cosmetic products has become a popular practice in many countries and high contents of formaldehyde in such products have been reported. In this study, a fluorescence methodology has been developed to measure the concentration of formaldehyde in detergents and cosmetic products. For sample preparation, a derivatization reaction by 2-methyl acetacetanlid is required. The formaldehyde content can then be quantified by photoluminescence method.The proposed method was applied for the analysis of Iranian brands of liquid detergents and cosmetic products. The formaldehyde content of these products was found to be in the range of 0.03–3.88%. Some brands of these products had higher concentration than the maximum allowed concentration of 0.2%.

## Figures and Tables

**Table 1:** Levels of independent variables established based on five-factor three-level design.

Variable	−1	0	+1
(*x*_1_) reaction time	5 min.	10 min.	15 min.
(*x*_2_) ethanol (% v/v)	10	20	30
(*x*_3_) ammonium acetate (M)	0.5	1.5	2.5
(*x*_4_) 2-methyl acetoacetanilid (M)	0.025	0.050	0.075
(*x*_5_) temperature	20	30	40

**Table 2:** Optimized parameters for derivatization reaction of formaldehyde.

Name of variable	Coded optimized values	Actual optimized values
Reaction time (*x*_1_)	−0.47	7.05
Ethanol (*x*_2_)	0.506	25.3
Acetate ammonium (*x*_3_)	0.72	2.2
2-Methyl acetoacetanilide (*x*_4_)	0.42	0.06
Temperature (*x*_5_)	−0.31	27

**Table 3:** Quantitative analysis of formaldehyde in detergents and cosmetic products.

Samples	Formaldehyde (%)
Dishwashing liquid 1	0.19
Dishwashing liquid 2	0.40
Dishwashing liquid 3	2.53
Hand washing liquid	ND
Hair shampoo 1	0.14
Hair shampoo 2	1.27
Baby shampoo	0.03
Body shampoo	0.25
Hand cleaner	3.88

**Table 4:** Recoveries of formaldehyde in raw material and cosmetic product samples.

Sample tested	Added formaldehyde mg·kg^−1^	Found formaldehyde mg·kg^−1^	Recovery factor (%)	RSD %
Surfactant	50.0	48.0	96.0	4.5
Hair shampoo	50.0	51.9	103.8	2.6
Body shampoo	50.0	47.7	95.4	3.4
Hand cleaner	50.0	45.9	91.8	2.4

All values are means (*n* = 3).

**Table 5:** Recoveries of formaldehyde in dishwashing liquid 1 and hair shampoo 1 samples with confidence limit 95%.

Sample^a^	Added formaldehyde (*µ*g·Kg^−1^)	Found formaldehyde (*µ*g·Kg^−1^)	Expected formaldehyde (*µ*g·Kg^−1^)	Recovery factor %	Relative errors %
Dishwashing 1 liquid	0	5.83 ± 0.2	—	—	—
	3	8.62 ± 0.4	8.83	97.62	−2.37
	5	11.13 ± 0.3	10.83	102.77	2.77
	8	13.48 ± 0.5	13.83	97.46	−2.53

Hair shampoo 1	0	4.88 ± 0.3	—	—	—
	3	8.26 ± 0.2	7.88	104.82	4.82
	5	9.50 ± 0.4	9.88	96.15	−3.84
	8	12.42 ± 0.6	12.88	96.42	−3.57

^a^All values are means (*n* = 3).

**Table 6:** Determination of formaldehyde in dishwashing liquid and hair shampoo in various times after production with confidence limit 95%.

Sample	Concentration after one week from production (ppm)	Concentration after one month from production (ppm)	Concentration after six months from production (ppm)	Concentration after nine months from production (ppm)
Dishwashing liquid 1	582 ± 0.2	542 ± 0.3	365 ± 0.4	216 ± 0.5
Hair shampoo 1	478 ± 0.5	438 ± 0.4	280 ± 0.5	155 ± 0.6

All values are means (*n* = 3).

**Table 7:** Tolerable concentrations of foreign species for determination of 1 × 10^−6^ M formaldehyde.

Foreign ion	Tolerable concentration (M)	Tolerable limit ((species)/(HCOH))	Relative error (%)
Na^+^, Cl^−^	1 × 10^−1^	100000	−2.7
Mg^2+^	1 × 10^−3^	1000	4.1
Ca^2+^	1 × 10^−3^	1000	−3.8
Benzaldehyde	1 × 10^−3^	1000	−2.3
Propionaldehyde	5 × 10^−4^	500	2.6
Acetaldehyde	5 × 10^−4^	500	2.9
Ni^2+^	1 × 10^−4^	100	3.6
Pb^2+^	1 × 10^−4^	100	−3.8
Cd^2+^	1 × 10^−4^	100	4.1
Fe^+3^	5 × 10^−5^	50	2.7

**Table 8:** The comparison between different methods.

Method	Temperature of derivative reaction	Detection limit (M)	Disadvantage of method	References
HPLC	25°C	—	Need organic solvent Cleanup step Time consuming	[8]
GC/MS	35°C and 10 min	1.3 × 10^−7^	Cleanup step	[12]
Spectrofluorimetry (acetyl acetone)	35°C and 1 hour	8.0 × 10^−9^	Need heating system Long reaction time	[24]
Spectrofluorimetry	25°C and 8 min	4.0 × 10^−9^	—	This work
