# Supplementary material for: Determination of Very Low Level of Free Formaldehyde in Liquid Detergents and Cosmetic Products Using Photoluminescence Method
Source: J Anal Methods Chem. 2016 Aug 21;2016:1720530. doi: 10.1155/2016/1720530 (PMC5011240; doi:10.1155/2016/1720530)
Supplement: Supplementary file 1 — Despite official regulations, the illegal use of formaldehyde in detergents and cosmetic products has become a popular practice in many countries and high contents of formaldehyde in such products have been reported. In this study, a fluorescence methodology has been developed to measure the concentration of formaldehyde in detergents and cosmetic products. For sample preparation, a derivatization reaction by 2-methyl acetacetanlid is required. The formaldehyde content can then be quantified by photoluminescence method.The proposed method was applied for the analysis of Iranian brands of liquid detergents and cosmetic products. The formaldehyde content of these products was found to be in the range of 0.03–3.88%. Some brands of these products had higher concentration than the maximum allowed concentration of 0.2%. [file 1720530.f1.pdf]

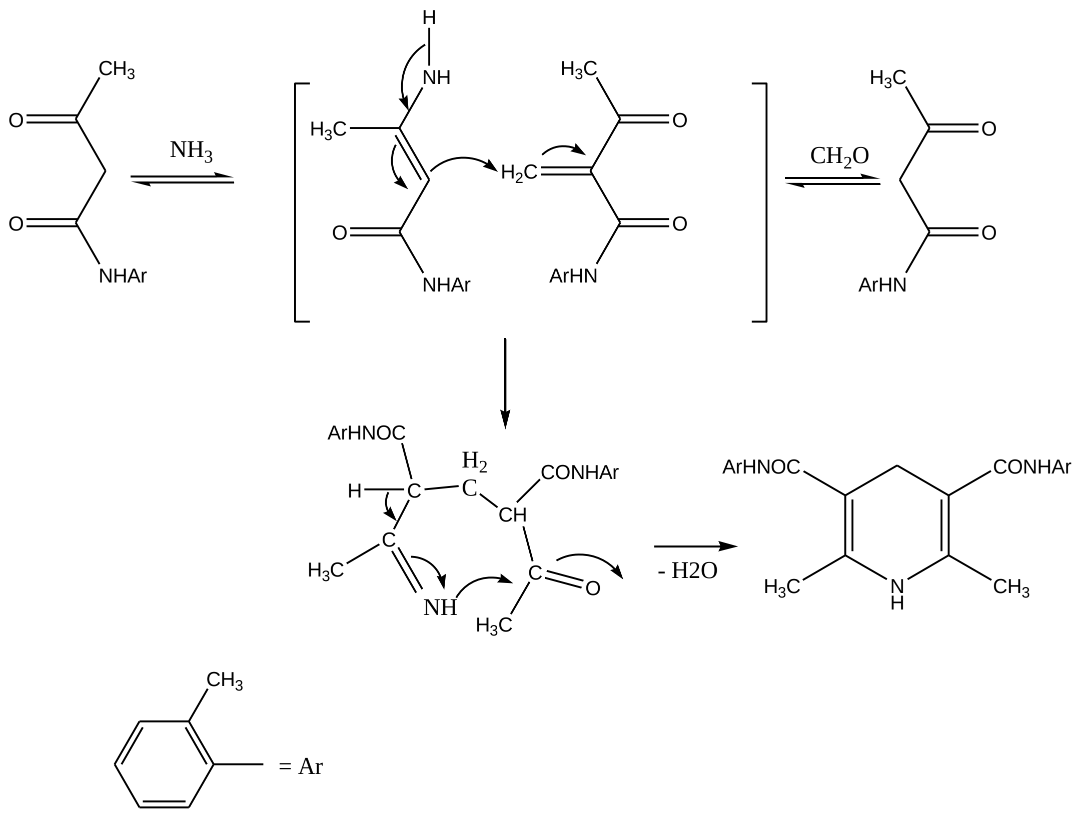

Scheme S1: Mechanism of hantzsch reaction of formaldehyde and 2-methyl acetoacetanilid

Figure captions

FIGURE S1: Response surface of the combined effect of reaction time, ethanol, acetate ammonium, 2-methylacetoacetanilid and temperature on derivitazation reaction of formaldehyde.

FIGURE S2: Counter plot of the combined effect of reaction time, ethanol, acetate ammonium, 2-methylacetoacetanilid and temperature on derivitazation reaction of formaldehyde.

FIGURE S3: 1H NMR product of formaldehyde with 2-methyl acetoacetanilid.

FIGURE S4: Multiplicity ^{1}H NMR from 1.5-5.5 ppm product of formaldehyde with 2-methyl acetoacetanilid.

FIGURE S5: Multiplicity ^{1}H NMR from 6.7-8.4 ppm product of formaldehyde with 2-methyl acetoacetanilid

FIGURE S6: Effect of temperature on fluorescence intensity for derivitazation reaction of 1×10^{-6} M formaldehyde with 2-methylacetoacetanilid

FIGURE S7: Effect of PH on fluorescence intensity for derivitazation reaction of 1×10^{-6} M formaldehyde with 2-methylacetoacetanilid

FIGURE S8: Three dimension flourescence spectrum of a shampoo sample after derivitaztation of formaldehyde

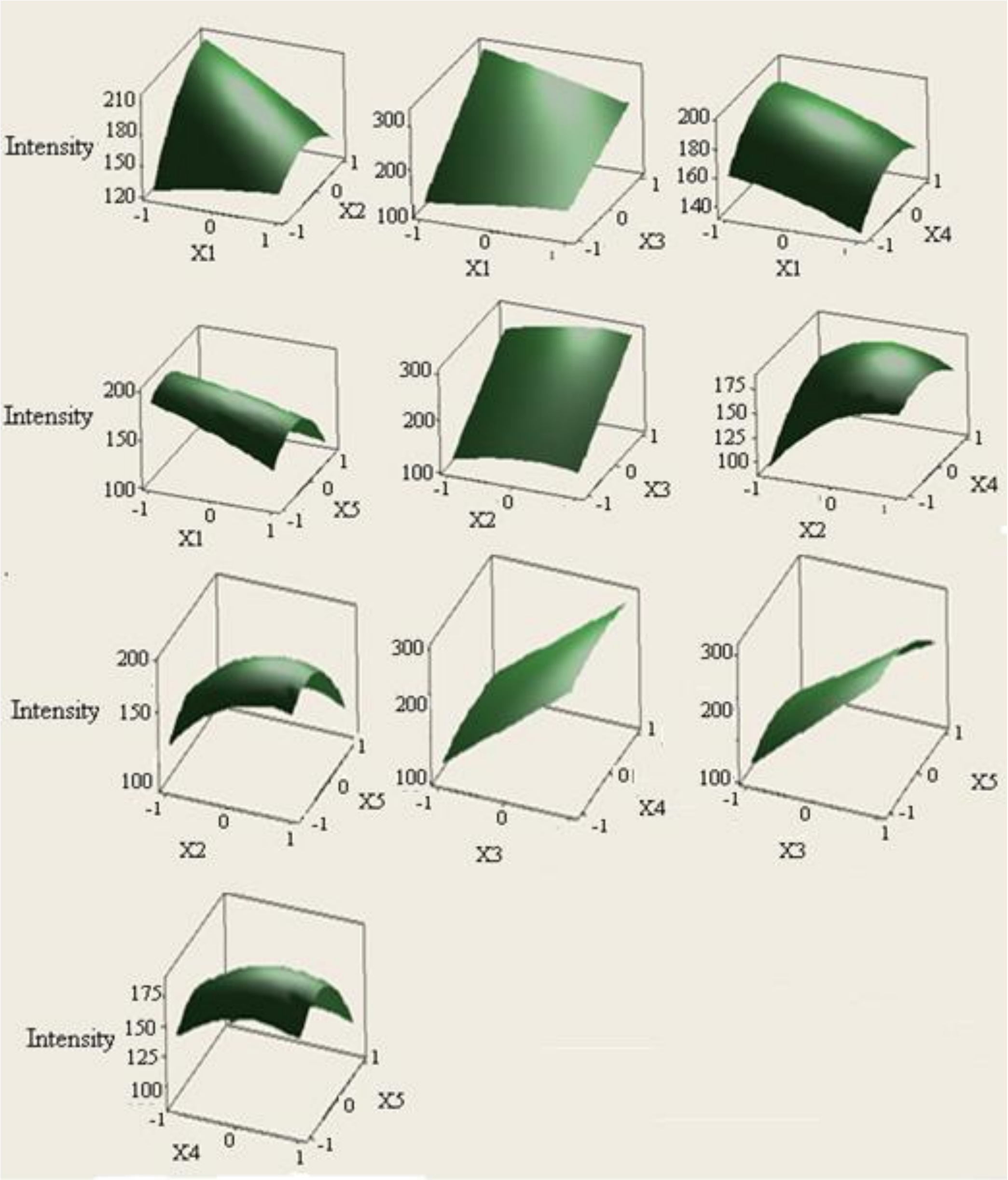

FIGURE S1

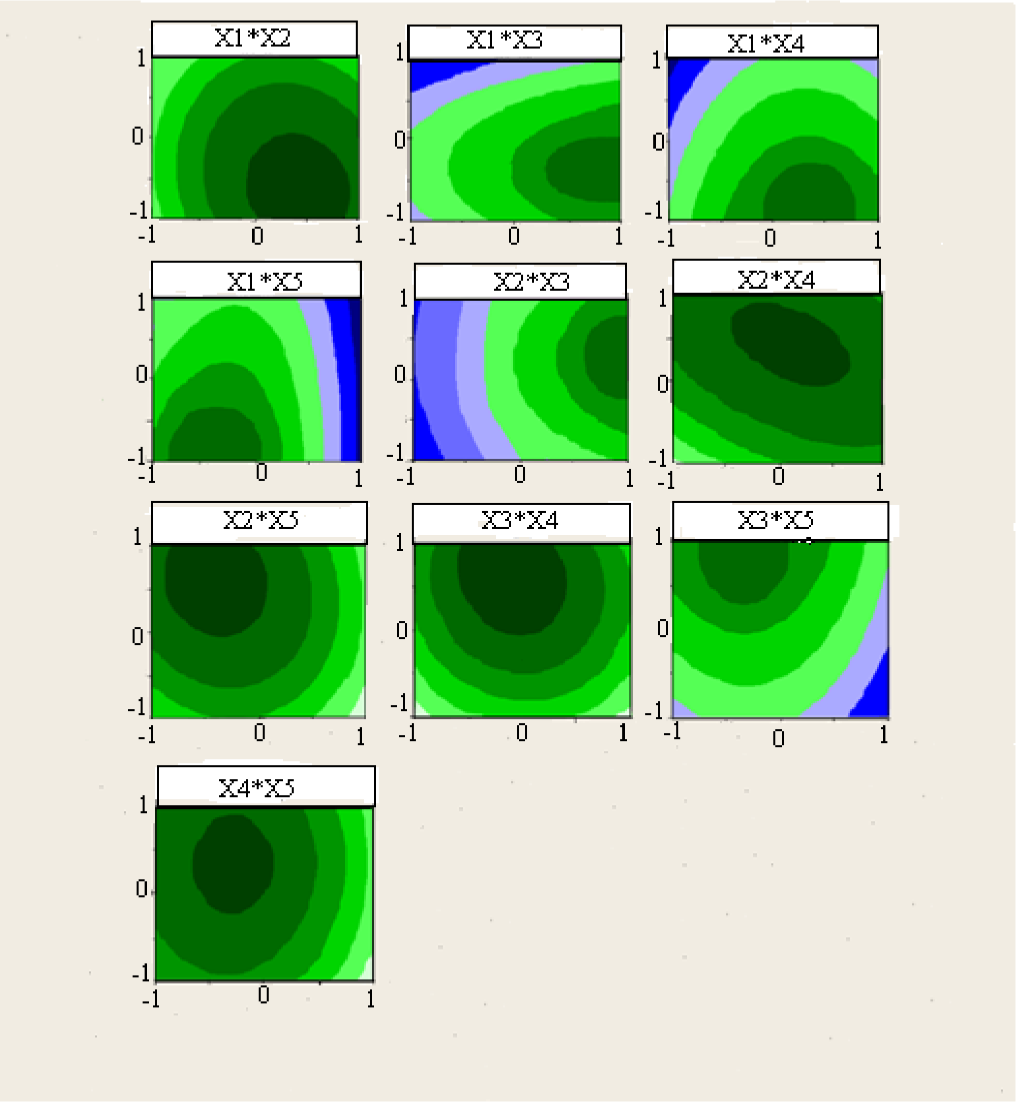

FIGURE S2

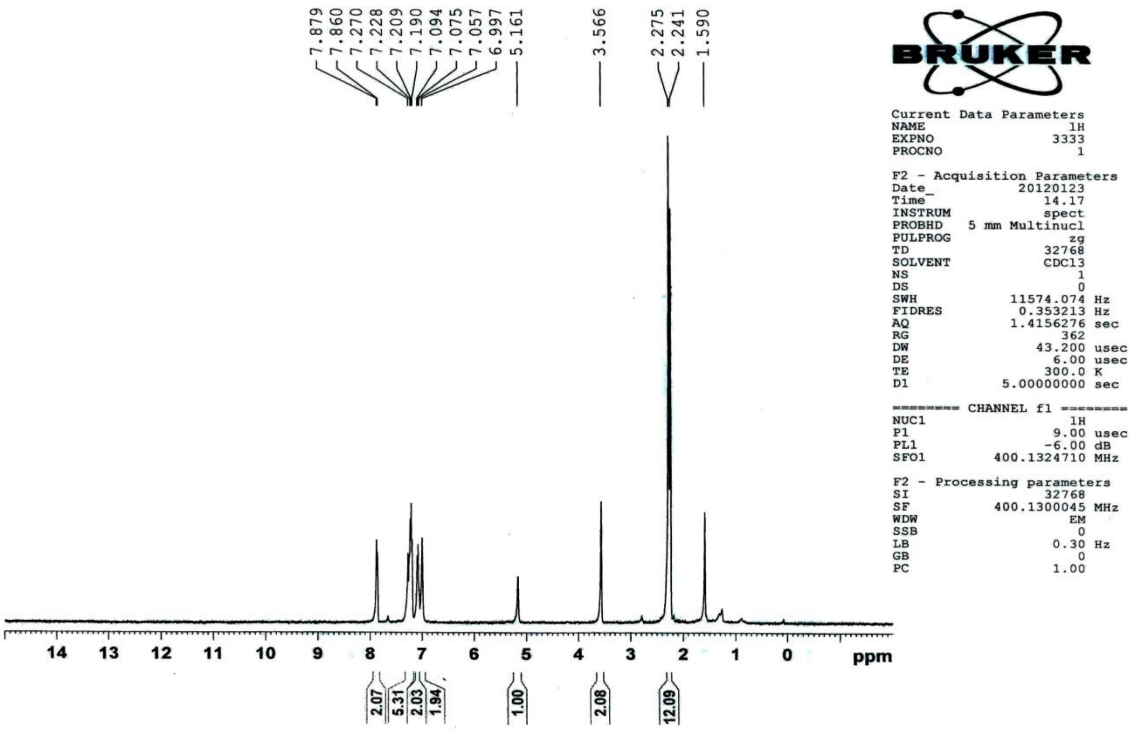
7.879
7.860
7.270
7.228
7.209
7.190
7.094
7.075
7.057
6.997
5.161
3.566
2.275
2.241
1.590
BRUKER
Current Data Parameters
NAME 1H
EXPNO 3333
PROCNO 1
F2 - Acquisition Parameters
Date_ 20120123
Time 14.17
INSTRUM spect
PROBHD 5 mm Multinucl
PULPROG zg
TD 32768
SOLVENT CDCl3
NS 1
DS 0
SWH 11574.074 Hz
FIDRES 0.353213 Hz
AQ 1.4156276 sec
RG 362
DW 43.200 usec
DE 6.00 usec
TE 300.0 K
D1 5.00000000 sec
======== CHANNEL f1 ========
NUC1 1H
P1 9.00 usec
PL1 -6.00 dB
SFO1 400.1324710 MHz
F2 - Processing parameters
SI 32768
SF 400.1300045 MHz
WDW EM
SSB 0
LB 0.30 Hz
GB 0
PC 1.00
14 13 12 11 10 9 8 7 6 5 4 3 2 1 0 ppm
2.07
5.31
2.03
1.94
1.00
2.08
12.09

FIGURE S3

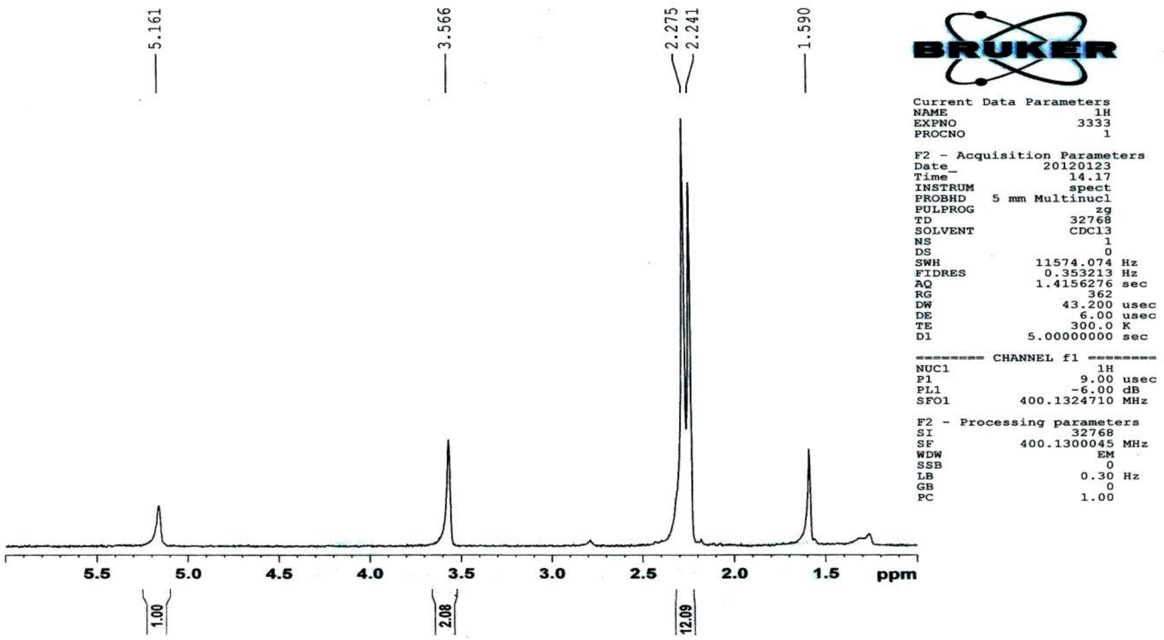
5.161
3.566
2.275
2.241
1.590
5.5 5.0 4.5 4.0 3.5 3.0 2.5 2.0 1.5 ppm
1.00
2.08
12.09
BRUKER
Current Data Parameters
NAME 1H
EXPNO 3333
PROCNO 1
F2 - Acquisition Parameters
Date_ 20120123
Time 14.17
INSTRUM spect
PROBHD 5 mm Multinucl
PULPROG zg
TD 32768
SOLVENT CDCl3
NS 1
DS 0
SWH 11574.074 Hz
FIDRES 0.353213 Hz
AQ 1.4156276 sec
RG 362
DW 43.200 usec
DE 6.00 usec
TE 300.0 K
D1 5.00000000 sec
======== CHANNEL f1 ========
NUC1 1H
P1 9.00 usec
PL1 -6.00 dB
SFO1 400.1324710 MHz
F2 - Processing parameters
SI 32768
SF 400.1300045 MHz
WDW EM
SSB 0
LB 0.30 Hz
GB 0
PC 1.00

FIGURE S4

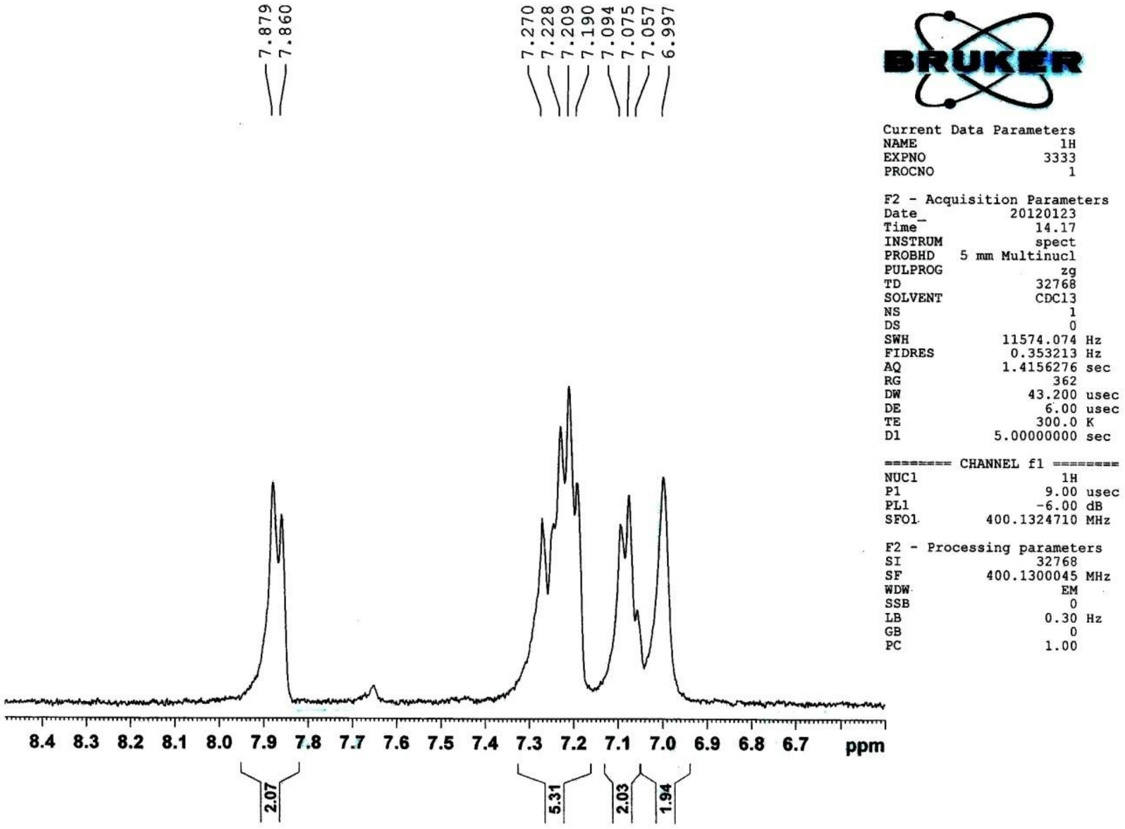
7.879
7.860
7.270
7.228
7.209
7.190
7.094
7.075
7.057
6.997
BRUKER
Current Data Parameters
NAME 1H
EXPNO 3333
PROCNO 1
F2 - Acquisition Parameters
Date_ 20120123
Time 14.17
INSTRUM spect
PROBHD 5 mm Multinucl
PULPROG zg
TD 32768
SOLVENT CDCl3
NS 1
DS 0
SWH 11574.074 Hz
FIDRES 0.353213 Hz
AQ 1.4156276 sec
RG 362
DW 43.200 usec
DE 6.00 usec
TE 300.0 K
D1 5.00000000 sec
======== CHANNEL f1 ========
NUC1 1H
P1 9.00 usec
PL1 -6.00 dB
SFO1 400.1324710 MHz
F2 - Processing parameters
SI 32768
SF 400.1300045 MHz
WDW EM
SSB 0
LB 0.30 Hz
GB 0
PC 1.00
8.4 8.3 8.2 8.1 8.0 7.9 7.8 7.7 7.6 7.5 7.4 7.3 7.2 7.1 7.0 6.9 6.8 6.7 ppm
2.07
5.31
2.03
1.94

FIGURE S5

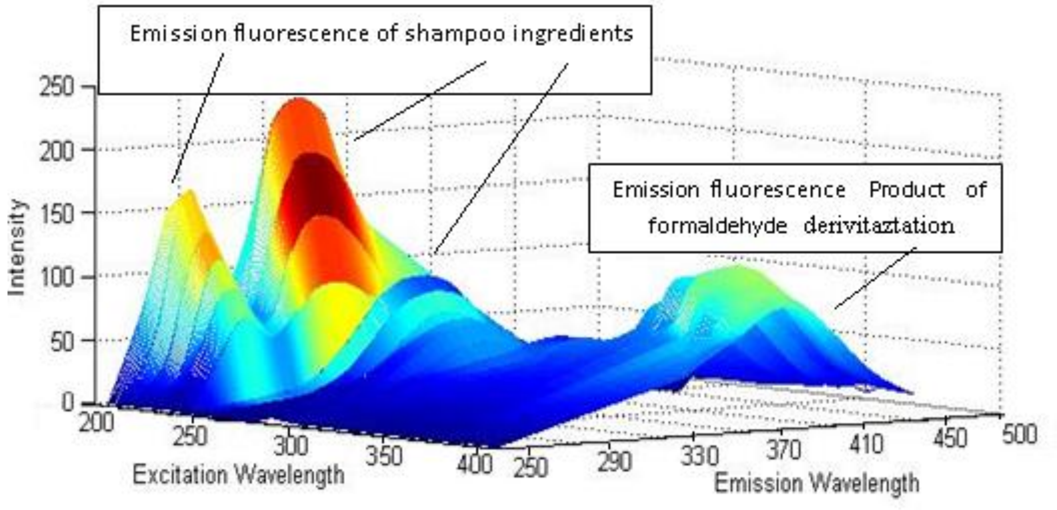
Emission fluorescence of shampoo ingredients
Emission fluorescence Product of formaldehyde derivitaztation
Intensity
250
200
150
100
50
0
200
250
300
350
400
Excitation Wavelength
250
290
330
370
410
450
500
Emission Wavelength

FIGURE S6

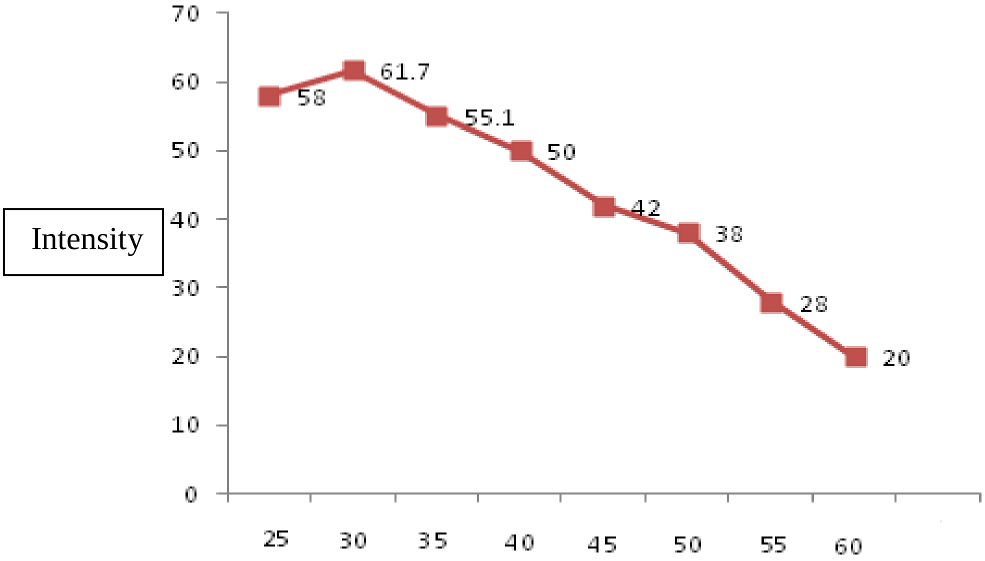
Intensity
70
60
50
40
30
20
10
0
61.7
58
55.1
50
42
38
28
20
25
30
35
40
45
50
55
60

FIGURE S7

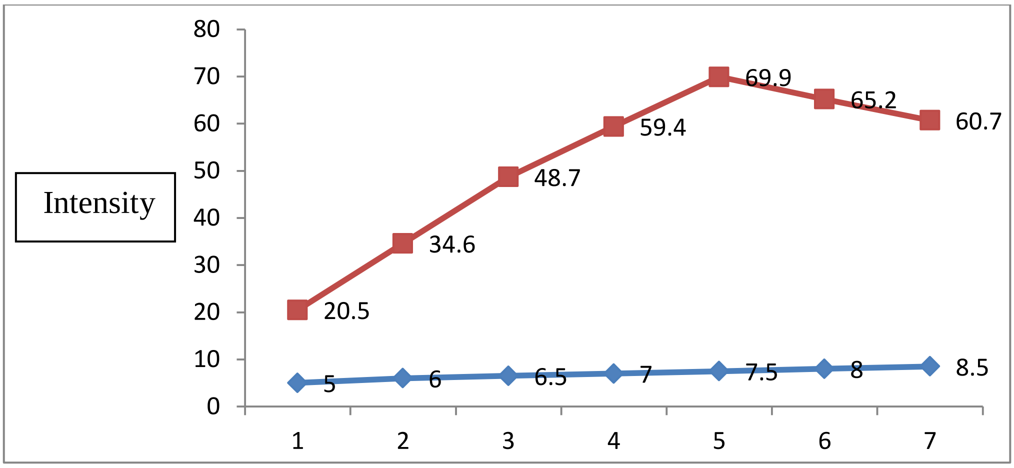
Intensity
80
70
60
50
40
30
20
10
0
1
2
3
4
5
6
7
20.5
34.6
48.7
59.4
69.9
65.2
60.7
5
6
6.5
7
7.5
8
8.5

FIGURE S8

TABLE captions

TABLE S1: Scheme of independent variables (Xi) for Box- Behnken design

TABLE S2: Estimated regression coefficients for derivitazation reaction of formaldehyde

TABLE S1: Scheme of independent variables (Xi) for Box- Behnken design

Std Order	Run Order	X_1	X_2	X_3	X_4	X_5
11	1	1	0	-1	0	1
30	2	0	0	1	0	-1
33	3	-1	0	0	0	-1
20	4	0	0	0	1	1
18	5	0	0	0	1	-1
3	6	-1	1	0	0	0
5	7	0	0	-1	-1	0
40	8	0	1	0	1	0
22	9	0	1	-1	0	0
34	10	1	0	0	0	-1
7	11	0	0	-1	1	0
26	12	1	0	0	-1	0
38	13	0	1	0	-1	0
23	14	0	-1	1	0	0
35	15	-1	0	0	0	1
37	16	0	-1	0	-1	0
10	17	0	1	0	0	-1
13	18	-1	0	-1	0	0
14	19	1	0	-1	0	0
43	20	0	0	0	0	0
1	21	-1	-1	0	0	0
24	22	0	1	1	0	0
31	23	0	0	-1	0	1
15	24	-1	0	1	0	0
29	25	0	0	-1	0	-1
32	26	0	0	1	0	1
28	27	0	-1	-1	0	0
2	29	1	-1	0	0	0
41	30	0	0	0	0	0
44	31	0	0	0	0	0
27	32	-1	0	0	1	0
36	33	1	0	0	0	1
39	34	0	-1	0	1	0
9	35	0	-1	0	0	-1
17	36	0	0	0	-1	-1
6	37	0	0	1	-1	0
19	38	0	0	0	-1	1
16	39	1	0	1	0	0
4	40	1	1	0	0	0
46	41	0	0	0	0	0
8	42	0	0	1	1	0
45	43	0	0	0	0	0
25	44	-1	0	0	-1	0
12	45	0	1	0	0	1
42	46	0	0	0	0	0

TABLE S2: Estimated regression coefficients for derivitazation reaction of formaldehyde

Term	Coef[a].	SE coef[b].	t[c]	p-value[d]
Constant	188.890	7.263	26.006	0.000
x_1	-29.086	4.448	-6.539	0.000
x_2	19.144	4.448	4.304	0.000
x_3	116.579	4.448	26.210	0.000
x_4	8.094	4.448	1.820	0.081
x_5	-11.167	4.448	-2.511	0.019
x_1*x_1	-38.974	6.023	-6.471	0.000
x_2*x_2	-22.639	6.023	-3.759	0.001
x_3*x_3	2.118	6.023	0.352	0.728
x_4*x_4	-25.145	6.023	-4.175	0.000
x_5*x_5	-20.514	6.023	-3.406	0.002
x_1*x_2	-21.202	8.896	-2.383	0.025
x_1*x_3	-22.313	8.896	-2.508	0.019
x_1*x_4	0.777	8.896	0.087	0.931
$x_1* x_5$	10.498	8.896	1.180	0.249
x_2*x_3	7.751	8.896	0.871	0.392
$x_2* x_4$	-39.969	8.896	-4.493	0.000
$x_2* x_5$	-20.762	8.896	-2.334	0.028
x_3*x_4	-1.809	8.896	-0.203	0.841
x_3*x_5	-13.945	8.896	-1.568	0.130
x_4*x_5	8.557	8.896	0.962	0.345
Adj-R square	97.19%			
Pred-R square	89.35%			
R square	94.94%			

a= coefficient, b=standard error of the coefficient, c=t test, d=p value
